# Would a robot trust you? Developmental robotics model of trust and theory of mind

**DOI:** 10.1098/rstb.2018.0032

**Published:** 2019-03-11

**Authors:** Samuele Vinanzi, Massimiliano Patacchiola, Antonio Chella, Angelo Cangelosi

**Affiliations:** 1 Cognitive Robotics Laboratory, The University of Manchester, Manchester, M13 9PL, UK; 2 Centre for Robotics and Neural Systems, University of Plymouth, Plymouth PL4 8AA, UK; 3 RoboticsLab, Università degli Studi di Palermo & ICAR-CNR, 90128 Palermo, Italy

**Keywords:** trust, theory of mind, episodic memory, cognitive robotics, developmental robotics, human–robot interaction

## Abstract

Trust is a critical issue in human–robot interactions: as robotic systems gain complexity, it becomes crucial for them to be able to blend into our society by maximizing their acceptability and reliability. Various studies have examined how trust is attributed by people to robots, but fewer have investigated the opposite scenario, where a robot is the trustor and a human is the trustee. The ability for an agent to evaluate the trustworthiness of its sources of information is particularly useful in joint task situations where people and robots must collaborate to reach shared goals. We propose an artificial cognitive architecture based on the developmental robotics paradigm that can estimate the trustworthiness of its human interactors for the purpose of decision making. This is accomplished using Theory of Mind (ToM), the psychological ability to assign to others beliefs and intentions that can differ from one’s owns. Our work is focused on a humanoid robot cognitive architecture that integrates a probabilistic ToM and trust model supported by an episodic memory system. We tested our architecture on an established developmental psychological experiment, achieving the same results obtained by children, thus demonstrating a new method to enhance the quality of human and robot collaborations.

This article is part of the theme issue ‘From social brains to social robots: applying neurocognitive insights to human–robot interaction’.

## 1. Introduction

The technological revolution taking place in the fields of robotics and artificial intelligence seems to indicate a future shift in our human-centred social paradigm towards a greater inclusion of artificial cognitive agents in our everyday environments. This means that collaborative scenarios between humans and robots will become more frequent and will have a deeper impact on everyday life. In this setting, research regarding trust in human–robot interactions (HRI) assumes a major importance in order to ensure the highest quality of the interaction itself, as trust directly affects the willingness of people to accept information produced by a robot and to cooperate with it. Many studies have already explored trust that humans give to robots and how this can be enhanced by tuning both the design and the behaviour of the machine, but not so much research has focused on the opposite scenario, that is the trust that artificial agents can assign to people. Despite this, the latter is a critical factor in joint tasks where humans and robots depend on each other’s effort to achieve a shared goal: whereas a robot can fail, so can a person. For an artificial agent to know when to trust or distrust somebody and adapt its plans to this prediction can make all the difference in the success or failure of the task.

Our work is centred on the design and development of an artificial cognitive architecture for a humanoid autonomous robot that incorporates trust, theory of mind (ToM) and episodic memory, as we believe these are the three key factors for the purpose of estimating the trustworthiness of others. We have tested our architecture on an established developmental psychology experiment [1] and the results we obtained confirm that our approach successfully models trust mechanisms and dynamics in cognitive robots.

## 2. Previous work

Trust is a fundamental, unavoidable component of social interactions that can be defined as the willingness of a party (the trustor) to rely on the actions of another party (the trustee), with the former having no control over the latter [2]. Moreover, to trust somebody to accomplish a specific task is to believe that he or she is reliable and committed towards the task. At the same time, distrust is not the mere the absence of trust but, instead, the belief that the other party is committed and at the same time non-reliable [3]. Trust is involved in every sort of social interaction and is a key factor in the achievement of successful relationships, in our personal safety [4] and in team cooperation [5].

The development of trust during childhood is still under debate by developmental psychologists. Erikson [6] theorized that infants not older than 2 years pass through a stage known as ‘trust versus mistrust’, where their propensity to trust is shaped by the quality of care received. This happens because infants highly depend upon the caregivers for sustenance and learn whether or not the latter regularly satisfy their basic needs, either learning that the world is a secure, trustable environment or an undependable, insecure place.

A psychological trait that relates to the mastery of one’s self trustfulness is ToM: the ability to attribute mental states to others (for instance beliefs, intentions and desires) that may differ from one’s own. In fact, the ability to correctly judge the trustworthiness of others is strongly correlated to the matureness of this trait [7] because a mature ToM allows one to perform behaviour prediction and provides clues on trustworthiness [8]. Despite ToM being universal in adults, the same cannot be said about preschoolers: while the latter are not completely lacking some form of ToM, this slowly develops with age [9,10]. An experiment conducted by Vanderbilt *et al*. [1] demonstrated that ToM matures around the fourth year of age and is completely developed by the fifth year.

Whereas trust is such an important factor in human interactions, it is also an essential component of HRI, in the sense that a great degree of trust improves the quality of interactions with the robot and, vice versa, successful interactions enhance the machine’s trustworthiness from the user’s point of view. Hawley [3] states that inanimate objects can be reliable but not genuinely trustworthy. This does not apply to humanoid robots, which are artificial agents that can communicate and interact and, by adopting the participant stance, can be worthy of trust or distrust (or neither) from their human partners [11]. In human and robot teaming scenarios, where the two share a common goal, trust is an essential component to successfully perform joint activities [12].

Our work is based on an established framework known as developmental robotics. Cangelosi *et al*. [13, p. 4] defined it as ‘the approach to the design of behavioural and cognitive capabilities in artificial agents that takes direct inspiration from the developmental principles and mechanisms observed in the natural cognitive systems of children’.

Whereas various studies have focused on trust in HRI contexts, with a human trustor and a robot trustee, our work aims to investigate the opposite, that is the level of trust assigned from a robot to its human partners. We state that a robot involved in a joint task with a human should be able to evaluate the trustworthiness of the latter and use this information to perform decision making. For example, in a hypothetical situation where the goal is to move some furniture around the house, it is equally important for both the robot and the human to trust the other’s ability to perform the job and eventually to dynamically adapt the plan. This can assume a greater importance in more critical scenarios, as in the performance of robotic-assisted surgery. Our aim is to design an artificial cognitive architecture able to evaluate the trustworthiness of the humans it interacts with, in order to predict their future behaviour. We believe that the key to such an architecture is the implementation of a ToM module in a robotic agent. Our work builds upon the research of Patacchiola & Cangelosi [14], who designed a Bayesian model that incorporates aspects of ToM and trust and applied it to the Vanderbilt experiment [1]. We took these findings a step further, expanding and incorporating them into a robotic system that can learn to distinguish trustworthy and untrustworthy sources of information and that can modify its behaviour according to its belief, thus remarking that it is possible to adopt a probabilistic approach to model and adapt ToM and trust in a unified scheme. Our system was able to reproduce the results obtained by Vanderbilt [1] on both mature and immature ToMs.

Lastly, we aimed in supporting this trust and ToM architecture with an episodic memory system that made it possible for the robot to make predictions about novel human informants with which it had never been familiarized. Episodic memory is a subcategory of the long-term declarative memory that stores memories about temporally dated episodes or events and temporal–spatial relations among them [15]. Knowledge of one’s personal history enhances the ability to accomplish several cognitive capabilities and is strictly related to the sense of self and consciousness. That is why many researchers have focused on the design of artificial episodic memory systems [16,17]. Our idea led us to the implementation of a system that is able to use memories of its past interactions to influence its behaviour towards someone with whom it had never interacted before. This means that the robot will develop a personal character, based upon the way it has been treated in the past, that will make it more or less keen to trust someone it does not know, as is the case with infants in the ‘trust versus mistrust’ phase theorized by Erikson [6]. This general and generic first impression towards novelty will then be subsequently reshaped by the interactions it will experience: for instance, if the robot distrusts someone who subsequently proves to be a helper, its behaviour will slowly change. We accomplished this by designing an algorithm inspired by the particle filter [18,19], a technique widely used for mobile robot localization.

## 3. Proposed method

The objective of our research is to implement an artificial cognitive architecture for a humanoid robot that incorporates a probabilistic unified trust and ToM module to evaluate the trustworthiness of human partners involved in joint tasks, paired with an episodic memory system. To reach our goal, we designed and developed a cognitive robotic system that is able to be subjected to a developmental psychology trial [1] and to obtain the same results as either an under or over 5-year-old child, therefore successfully simulating the children’s cognitive abilities and ToM immatureness or matureness, respectively.

### (a) Theoretical background

The psychological test we aimed to reproduce is the one designed by Vanderbilt [1], in particular, experiment number 1: 90 preschool-age children, equally divided into 3-, 4- and 5-year-olds, were shown a video in which an adult actor, either a helper or a tricker, gave advice to another adult who was trying to locate a sticker hidden in one of two boxes. Helpers would suggest the correct location, whilst the trickers always pointed to the wrong box. Subsequently, that same pointer would give some advice to the child on the same sticker-finding task and the participant would decide whether to follow that suggestion or not. Based on the children’s choices and on some meta-cognitive questions submitted to them, Vanderbilt theorized that only the 5-year-olds were able to differentiate the helpers from the trickers, therefore demonstrating the possession of a mature ToM.

**Figure 1.**
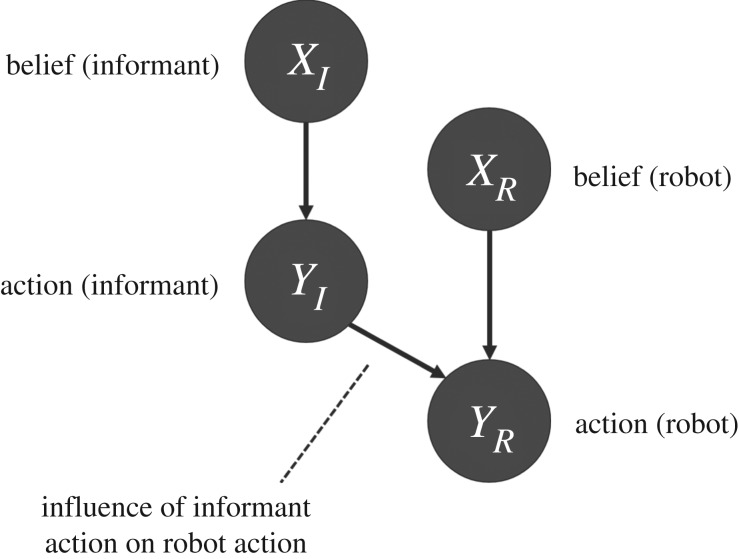
The BN that models the relation between the robot and an informant. The agent generates a separate network for each user, with the same structure but different probability distribution.

In order to substitute one of the preschoolers with a humanoid robot, the latter needs to possess a trust and ToM computational model to be able to predict the intentions and beliefs of the pointers with which it is going to interact. A good candidate for this model is the developmental Bayesian model of trust designed by Patacchiola [14], which uses a probabilistic approach to solve the problem of trust estimation. Bayesian networks (BNs) are probabilistic graphical models that represent conditional dependencies between a set of random variables. This particular model uses discrete Boolean variables that assume two states: *a* and *b*, each corresponding to one of the two positions where the stickers can be located in the experiment. A graphical illustration of this BN can be observed in figure 1: the two nodes *X*_*R*_ and *Y*_*R*_ represent, respectively, the beliefs and actions of the robot. The posterior distribution of the node *Y*_*R*_ allows the agent to choose the action to perform: that means searching for the sticker in position *a* or *b*. The connection between *Y*_*I*_ and *Y*_*R*_ represents the influence that the opinions of the informant have on the agent’s action. The action of the agent is then a consequence of its own belief *X*_*R*_ and the informant action *Y*_*I*_. Lastly, the estimation of *X*_*I*_, the informant’s belief, makes the agent able to effectively discriminate a trickery from a non-malevolent human error. The cognitive architecture we designed creates one of these BNs for each informant it interacts with and uses it to predict its future behaviour.

This developmental Bayesian model has been extended with an episodic memory system that will be described more in detail in §3d.

### (b) Cognitive system architecture

**Figure 2.**
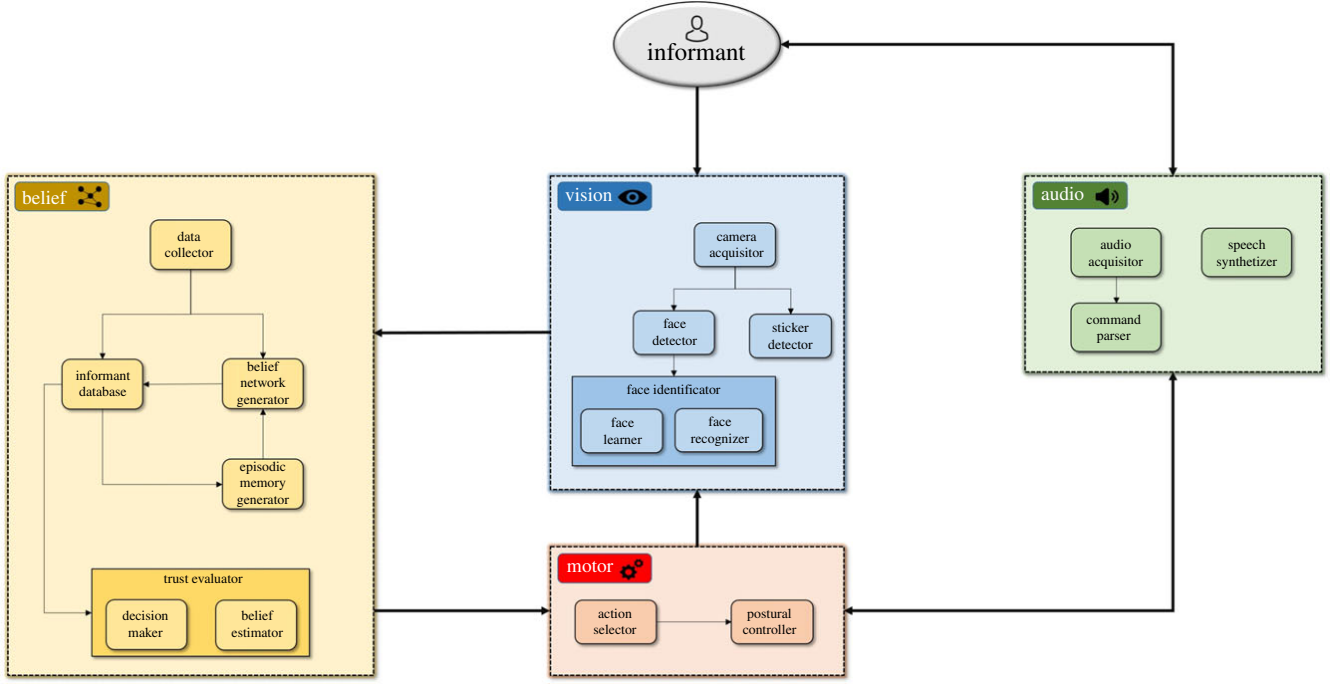
Architecture of the artificial cognitive agent. The human informant interacts with the robot through the vision and audio modules, which, respectively, perform image processing (face detection and recognition) and vocal command parsing. Data then flows to the motor module in charge of the robot’s joints, and to the belief module that manages the collection of BNs memorized by the agent. (Online version in colour.)

An overview of our robotic architecture is shown in figure 2. This cognitive system interfaces with each informant individually and performs various perception and actuation tasks. The audio module is used to synthesize vocal outputs to guide the users through the course of the experiment and to process vocal commands. The motor module is responsible of piloting the robot’s joints and controlling its head and body movements. The vision module is in charge of face detection and recognition through Haar Cascade [20] and Local Binary Pattern Histogram [21] machine learning algorithms and also to detect the presence of the sticker on the table. The belief module is the core component that makes the robot able to evaluate the trustworthiness of the informant who has been recognized, eventually using its episodic memory, to generate a new BN ‘on the fly’ in order to react to a novel user.

### (c) Belief

The belief unit encircles all the algorithms that deal with the Bayesian belief networks. One of its functions is to store episodes, which are data structures that encode sticker searching events inclusive of both the sticker position and the suggestion received from the informer. The way in which these data are created depends on the agent’s ToM matureness: in the case of a misleading suggestion, the immature agent associates the action *Y*_*I*_ to the wrong belief *X*_*I*_, whereas the agent with mature ToM identifies the deception and recognizes that *Y*_*I*_ = ¬*X*_*I*_. Because of this deficit in reading the informant’s intention, the agent with immature ToM collects wrong statistical data that will distort inference in subsequent phases.

Once the agent has collected a certain amount of episodes from an informant, it can generate a BN associated with him or her using the maximum-likelihood estimation (MLE) algorithm [22] to determine the conditional probability tables of its nodes. For the root nodes *X*_*I*_ and *X*_*R*_ we calculate these probabilities as:

3.1 $$\begin{array}{rl} & P_{Y}(a)=\theta \\ \text{and}\; & P_{Y}(b)=1-\theta . \end{array}\}$$

Denoting *N*_*a*_ and *N*_*b*_ as the number of times the pointer chooses *a* or *b*, we can estimate *θ* as:

3.2 $$\;\hat{\;\theta }=\frac{N_{a}}{N_{a}+N_{b}}.$$

For the nodes *Y*_*I*_ and *Y*_*R*_, instead, we have to also take into consideration the influence of the parents.

Once a BN has been created for a certain user and its parameters have been learned from the interactions, it is possible to infer the posterior probability of the nodes given some observations. In particular, we are interested in estimating the belief given an action and vice versa. We calculate posterior distributions using Pearl’s Message-Passing algorithm [23].

### (d) Episodic memory

The ability to use one’s own past memories to take decisions in the present and future is an important skill that enhances cognitive processes. An implementation of such skill would enable the robot to react reasonably towards a novel informant with whom it has never been familiarized. On a technical level, the main problem is to generate on the fly a new BN with adequate parameters to use with that unknown informer. These parameters will depend upon the robot’s personal character built in respect of the way it has been treated in the past: an agent who has been tricked often would learn to be mistrustful and vice versa, as in the ‘trust versus mistrust’ phase in child development [6].

The design guidelines that we followed in the creation of our algorithm were the following: memories fade away with time; the details become blurred proportionally to the amount of memories possessed; shocking events such as surprises and betrayals should be more difficult to forget than ordinary, expected experiences.

Our algorithm draws inspiration from the particle filter technique widely used in mobile robot localization [18]. Whenever an unknown informant is met, this component generates on the fly a certain number of episodes to train a new BN.

We define the set of BNs memorized by the agent as:

3.3 $$S=[s_{0},s_{1},\ldots ,s_{n}],$$

where *n* is the number of informants known by the agent.

Each BN *s*_*i*_ was generated by a set of episodes, and these are going to be denoted as *replay datasets* for that BN:

3.4 $$E_{s_{i}}=[\varepsilon_{0}^{\left(s_{i}\right)},\varepsilon_{1}^{\left(s_{i}\right)},\ldots ,\varepsilon_{m}^{\left(s_{i}\right)}]\;:\;s_{i}\in S,$$

where *m* is equal to the number of episodes of the replay dataset. So, in this notation $\varepsilon_{\;j}^{\left(s_{i}\right)}$ represents the *j*th episode of the replay dataset that formed the BN *s*_*i*_.

The equation we are about to introduce uses information theory to quantify the amount of information each specific episode represents. Our goal is to find how much this value differs from the total entropy of its replay dataset: a high difference means that the event is to be considered surprising and must be easier to recall than ordinary, unsurprising events. For example, if an informant who is always been trustful suddenly tricks the agent, this betrayal will be remembered with a greater impact. At the same time, all of the memories are subject to a progressive time degradation that tends to blur them with the passage of time, dependent on their importance.

Formally, a real factor denoted as importance value *v* defined in the interval [0, 1] is calculated for every episode $\varepsilon_{\;j}^{\left(s_{i}\right)}$ as the difference between the amount of information of the episode, $I(\varepsilon_{\;j}^{\left(s_{i}\right)})$ and the total entropy of its replay dataset, $H(E_{s_{i}})$, divided by the discrete temporal difference from the time when the memory was formed.

3.5 $$\begin{array}{rl} v(\varepsilon_{\;j}^{\left(s_{i}\right)}) & =\frac{|I(\varepsilon_{\;j}^{\left(s_{i}\right)})-H(E_{s_{i}})|}{\Delta t+1}\\ & =\frac{|-\log_{2}P(\varepsilon_{\;j}^{\left(s_{i}\right)})+\sum_{\varepsilon \in E_{s_{i}}}P(\varepsilon )\log_{2}P(\varepsilon )|}{t_{\mathrm{present}}-t_{\varepsilon_{\;j}^{\left(s_{i}\right)}}+1}. \end{array}$$

Once *v* has been calculated it can be used to determine a replication factor by projecting it on a step function defined as:

3.6 $$F(v)=\left\{ \begin{array}{rl} 0 & \;\text{if}\;0\leq \;v\leq \;0.005\\ 1 & \;\text{if}\;0.005<\;v\leq \;0.3\\ 2 & \;\text{if}\;0.3<\;v\leq \;0.6\\ 3 & \;\text{if}\;0.6<\;v\leq \;1. \end{array}\right.$$

Every episode from each replay dataset in the agent’s memory is replicated *F*(*v*) times.

**Figure 3.**
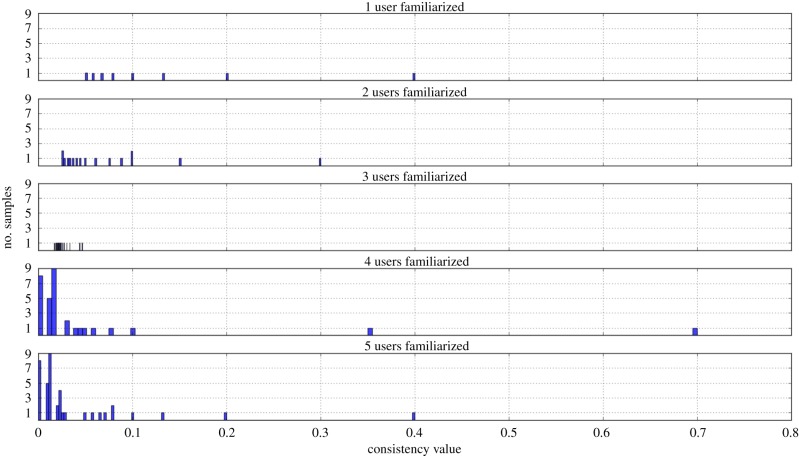
Consistency values histogram of the episodes memorized by the agent through progressive interactions with different informants (from 100% helpful to 100% tricky at 25% steps of variation. The opposite, that is from 100% tricky to 100% helpful, produces the same graph). (Online version in colour.)

The thresholds of the step function have been defined by observing the probability distribution of the importance value *v* obtained by making the robot interact with five different informants, the latter characterized by the following percentages of helpful interactions: 100%, 75%, 50%, 25% and 0%. This distribution is observable in figure 3. Most samples tend to fall in the range [0, 0.3], so samples that are contained in this interval are kept as they are, with no replications. Samples included in the intervals (0.3, 0.6] and (0.6, 1] are the most surprising ones for the agent and get, respectively, duplicated and triplicated. The bottom 5% of the domain, i.e. *v* values less than or equal to 0.005, are discarded (they are forgotten).

The next step in our algorithm is to pick *k* episodes to form the replay dataset for the new BN we intend to create, $E_{s_{n+1}}$. It is possible to select random samples but this will lead to poor final results, so we instead operate a systematic resampling [24], a kind of weighted random sampling. To avoid biases dependent on the two positions in which the sticker can be located, we want the agent to distinguish only between positive and negative actions, or ‘truth’ and ‘lies’: for this reason, instead of picking *k* samples the system will only select *k*/2 and for each of them it will generate the corresponding symmetric example. For instance, if a {*sticker in *a*, correct suggestion*} episode is sampled, a {*sticker in *b*, correct suggestion*} episode will also be included in the new replay dataset.

**Figure 4.**
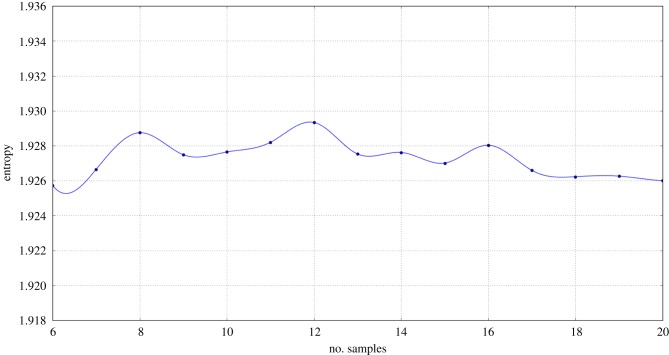
Mean entropy of episodic memory networks generated with different numbers of samples. Given the random component intrinsic in the algorithm, a very large number of samples (10^5^) have been generated for every value of *k*. (Online version in colour.)

The optimal number of samples *k* has been investigated: a low value would result in a gullible belief network, whilst a high value would make it stubborn to changes. Our goal was to let the robot possess a firm but changeable prejudice about the novel informant. By analysing the mean entropy of the episodic networks generated by different values of *k*, as shown in figure 4, we selected *k* = 10 for the following reasons: it is an even number, so it will produce an integer *k*/2 value, it is neither too low or too high to incur the above-mentioned problems and, finally, it is a local minimum of entropy.

Finally, MLE is applied to the new reply dataset to evaluate the parameters of the network. This new BN will be stored in the agent’s long-term memory as *s*_*n*+1_ and will be used to predict the trustworthiness of the new informant.

### (e) Evaluation criteria for belief networks

We introduce the trust factor *T* as a measure of how keen a BN is to trust the informant.

To calculate *T*, we hypothetically imagine that the sticker is located in position *a* and execute a belief estimation task, as described in §4b(iii), to obtain the posterior probability *p* of node *Y*_*I*_: what we are doing is computing the probability that the informant will give correct advice given the matureness of the agent’s ToM. At this point, the value is scaled in a [ −1, +1] interval, where the two endpoints −1 and +1 represent, respectively, complete trickers and helpers, which means BNs whose parameters have been computed from replay datasets containing only truthful or untruthful episodes. Values of *T* in between represent informants that are partially helpful and misleading. To perform the scaling we required the maximum and minimum values of *p* and we found them by building two BNs formed by a large (10^4^) number of, respectively, helpful and misleading episodes and computed *p* for each of them, resulting in *p* = 0.75 for the helper network and *p* = 0.25 for the tricker one. For a generic interval [*a*, *b*], the scaling is computed as:

3.7 $$T(p)=\frac{\left(b-a)(p-\min\right)}{\max-\min}+a,$$

with min = 0.25, max = 0.75, *a* = −1 and *b* = +1.

Equation (3.7) is used in §5 to evaluate the experimental data collected on experiments and simulations.

While experiencing new interactions, the BN can acquire new statistical data and adapt its behaviour over time. This happens when *T* changes sign, that is when the number of negative interactions surpasses the positive ones, or vice versa.

## 4. Experiments

### (a) Experimental set-up

For our experiments, we used a Softbank Pepper, a humanoid social robot designed to operate in human environments. A single table is present in the environment, on top of which a printed mat, depicting the two positions *a* and *b* and some instructions for the participants, are laid down. The robot is located on one side of the table, while the informants take turns in sitting in front of it, on the opposite side of the desk. The participants are provided with a sticker that they are able to move between the left and right locations. Each informant is instructed to act as a helper, always revealing the correct position of the sticker, or a tricker, always giving wrong advice.

### (b) Procedure

As for the original experiment by Vanderbilt [1], our trial is divided in three sections: familiarization, decision making and belief estimation. Having already introduced the technical details of the cognitive architecture, this section will focus on describing the logical flow of operations.

#### (i) Familiarization phase

**Figure 5.**
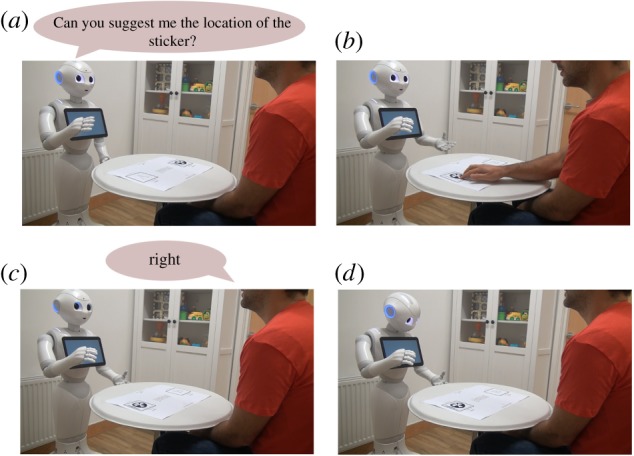
Familiarization phase with a tricker informant. (*a*) The robot asks for a suggestion on the sticker’s location. (*b*) The informant places the sticker in one of the two positions. (*c*) The informant gives its suggestion on where to find the sticker. Note how the tricker gives misleading directions. (*d*) The robot searches for the presence of the sticker in the suggested position and records the episode. (Online version in colour.)

The aim of the first phase is to make the robot familiarize with the informants, which means learning the correct parameters of the BNs associated with them. A visual description of the process is shown in figure 5.

One at a time, each of the informants sit at the table while the robot captures some face images to be able to recognize them in the future. The user is given time to place the sticker on one of the two positions marked on the table, *a* or *b*. After that, the robot asks for a suggestion on the location of the above-mentioned sticker and, once received, it follows this suggestion blindly, searching for the marker. Based on of the result of this detection and on the matureness of the robot’s ToM model, an episode is created in its memory.

Following the original experiment, this demonstrative task is repeated six times per user, with the sticker located 50% of the time on position *a* and the other 50% of the time on position *b*. At the end of this procedure, the data acquired are used to build a BN for that informant, as described in §3c.

After the familiarization phase the robot possesses a BN for every known informant.

#### (ii) Decision making phase

**Figure 6.**
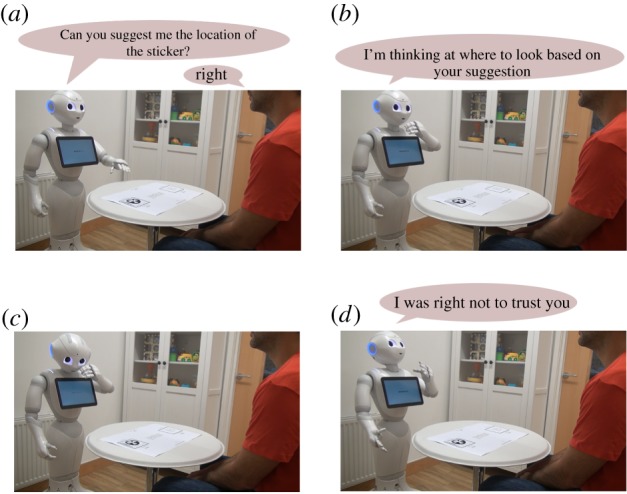
Decision-making phase with a tricker informant. (*a*) The robot asks for a suggestion on the location of the sticker and receives a misleading suggestion from the informant. (*b*) The robot performs inference on that informant’s belief network. (*c*) The agent decides that the informant will probably try to trick it, so it looks in the opposite location. (*d*) The robot finds the sticker and gives feedback to the informant. (Online version in colour.)

In the decision making phase, shown in figure 6, the robot has to correctly locate the sticker, choosing one of the two locations given the informant’s opinion. Initially, one informant sits at the table: if he or she is identified, the associated BN is fetched and used in the subsequent computations, otherwise, a new one is generated on the fly for him or her using episodic memory.

At this point, the user positions the sticker and gives a suggestion. The agent performs inference on the BN in order to calculate the posterior probabilities given *Y*_*I*_ as evidence. In particular, if $P_{Y_{R}}(a)>P_{Y_{R}}(b)$ the robot will investigate position *a* and if $P_{Y_{R}}(a)<P_{Y_{R}}(b)$ it will look at position *b*.

The episode generated by this interaction will be used to update the parameters of the BN, making the robot progressively adapt to the user’ behaviour.

#### (iii) Belief estimation phase

In the original trial, children where asked some meta-cognitive questions in order to investigate their perception of the informants and to examine their ToM matureness. To test the same on the artificial cognitive agent, Bayesian inference can be used.

**Figure 7.**
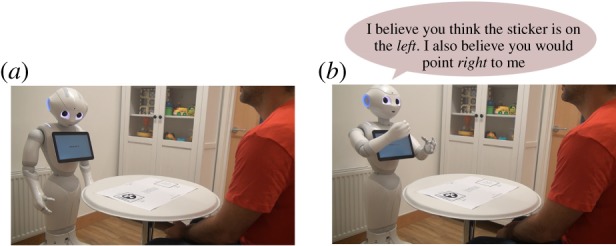
Belief estimation phase with a tricker informant. (*a*) The robot recognizes the informant using machine learning techniques and looks at the table to find the position of the sticker. (*b*) The robot computes inference on the informant’s belief network and predicts what he would suggest in that situation. (Online version in colour.)

The belief estimation phase is very similar to the decision making one and differs only for the kind of inference computed. The robot uses its face detection and recognition algorithms to identify the informant with whom it is interacting, either a known or an unknown one, then it observes the table to identify the position of the sticker. Setting *X*_*R*_ and *Y*_*R*_ as evidence, the Message Passing algorithm [23] is used to calculate the posterior probabilities for the rest of the network. At this point, the agent can use the probability distributions in nodes *X*_*I*_ and *Y*_*I*_ to infer the informant’s belief and the location that most probably would have been suggested by him or her. This process is shown in figure 7.

### (c) Simulations on character formation

In order to test the effects of episodic memory on character formation, we made a mature ToM simulated agent familiarize with different sets of informants to study how it would subsequently react to a novel person. Each set was composed of eight informants. In particular, the first set was formed by eight helpers, the second by six helpers and two trickers and so on until the last set was made up of eight trickers. For each set of informants familiarized, 100 episodic belief networks were generated and their trust factors *T* were computed using equation (3.7) and plotted on a histogram. After each set was processed, the memory of the robot was reset so that the effects of each group of informants could be analysed individually.

## 5. Results and discussion

### (a) Trust and theory of mind

The final results of Vanderbilt [1] showed that only the children with mature ToM distinguished between helpers and trickers, thus confirming the fact that children’s reasoning about whom to trust is directly correlated with their understanding of mental life. Our research is coherent with these results: when the robot is provided with a mature ToM model, it is able to correctly recognize realiable sources from unrealiable ones, accepting suggestion from the former whilst rejecting it from the latter. By contrast, if the agent is operated with an immature ToM model, it will fail in the evaluation. To understand why this happens, we will examine the posterior distributions in each node of a BN after the inference that takes place in the decision making and belief estimation phases.

Table 1 shows the results obtained from the interaction of a helper and a tricker with two agents with, respectively, a mature and an immature ToM.

**Table 1.** BN node values for both a mature ToM (1) and an immature ToM (2) agent. The table rows represent the posterior probability distributions associated with each node of the networks, for both decision making (DM) and belief estimation (BE) tasks.

mature ToM										immature ToM							
	DM		BE		DM		BE			DM		BE		DM		BE	
	*a*	*b*	*a*	*b*	*a*	*b*	*a*	*b*		*a*	*b*	*a*	*b*	*a*	*b*	*a*	*b*
*X*_*R*_	0.5	0.5	1.0	0.0	0.5	0.5	1.0	0.0	*X_R_*	0.5	0.5	1.0	0.0	0.5	0.5	1.0	0.0
*Y*_*R*_	**0.65**	0.35	1.0	0.0	0.35	**0.65**	1.0	0.0	*Y*_*R*_	**0.65**	0.35	1.0	0.0	**0.65**	0.35	1.0	0.0
*X*_*I*_	0.8	0.2	**0.57**	0.43	0.2	0.8	**0.57**	0.43	*X*_*I*_	0.8	0.2	**0.57**	0.43	0.8	0.2	**0.57**	0.43
*Y*_*I*_	1.0	0.0	**0.62**	0.38	1.0	0.0	0.38	**0.62**	*Y*_*I*_	1.0	0.0	**0.62**	0.38	1.0	0.0	**0.62**	0.38
helper						tricker			helper			tricker					
(1)										(2)							

In the decision-making task, the helper indicated position *a* when the sticker was placed in that location. The suggestion was accepted by both the agents, as demonstrated by the inequality $P_{Y_{R}}(a)>P_{Y_{R}}(b)$. The behaviour of the agents differed towards the tricker who, in contrast, suggested position *a* when the sticker was located in *b*. The mature agent rejected the suggestion and this happened because $P_{Y_{R}}(a)<P_{Y_{R}}(b)$, which means that the robot decided to look at the other location on the table. The immature agent could not recognize the deception and accepted the misleading advice, as observable in the posterior distribution $P_{Y_{R}}(a)>P_{Y_{R}}(b)$, thus falling in with the deception.

During the belief estimation task, with the sticker placed in *a* and a helpful informant, both of the agents output $P_{X_{I}}(a)>P_{X_{I}}(b)$ and $P_{Y_{I}}(a)>P_{Y_{I}}(b)$, thus correctly anticipating the helper’s mental states. When facing the tricker, instead, the mature agent correctly predicts the malevolent intentions by outputting $P_{X_{I}}(a)<P_{X_{I}}(b)$ and $P_{Y_{I}}(a)<P_{Y_{I}}(b)$, while the immature agent failed to do so by predicting $P_{X_{I}}(a)>P_{X_{I}}(b)$ and $P_{Y_{I}}(a)>P_{Y_{I}}(b)$.

### (b) Episodic memory

**Figure 8.**
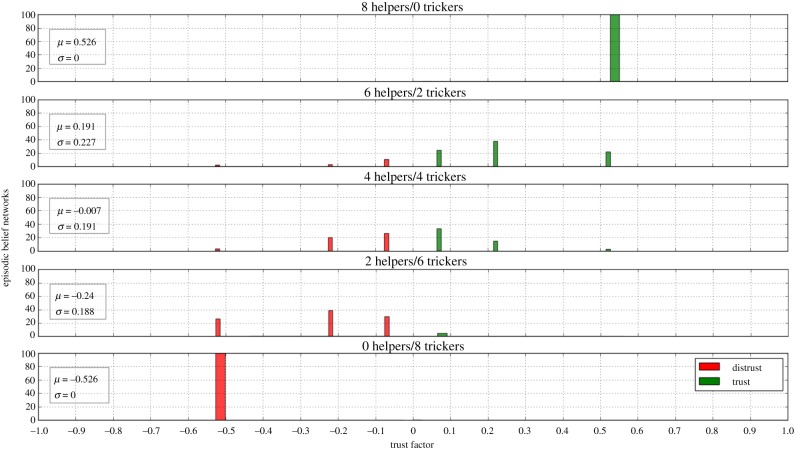
Reliability histogram of episodic belief networks generated by agents possessing different histories of interactions. Green bars represent trustful BNs (*T* > 0) and red bars depict BNs that tend to distrust (*T* < 0). Agents that have a more positive than negative background tend to be more prone to trust a new informant and vice versa. When *T* = 0, the informant is neither trusted nor distrusted and the agent will act randomly. (Online version in colour.)

Given the non-deterministic nature of the algorithm used to generate episodic belief networks, a more statistical method of evaluation is needed to report the results of this module. The histograms of the trust factors *T* obtained with the procedure described in §4c can be interpreted as the different characters that emerge in the robot, which means the tendency it has to trust or distrust a novel informant based on the interactions it has been faced with in the past. So, as shown in figure 8, an agent who is used to be tricked most of the time will tend to distrust somebody it meets for the first time, whereas a robot that has been treated kindly will learn to trust people and tend to consider them trustworthy until presented with contrary evidence. This behaviour mimics exactly the ‘trust versus mistrust’ stage of infancy theorized by Erikson [6], in which children learn to shape their personality by succeeding or failing in developing trust based on the quality of care received during infancy.

## 6. Conclusion and future work

In this paper, we discussed an artificial cognitive architecture for trust, ToM and episodic memory in a HRI scenario that can enhance the performance of artificial agents in shared goal contexts. We have extended the previous work by Patacchiola [14] by integrating the original model into a complete robotic architecture and extending it with an episodic memory component that enables it to remember and make use of its past experiences to develop a personal character and, doing so, to improve its cognitive abilities. We designed an artificial agent that is able to interact with the world around it and estimate the trustworthiness of its information sources to make autonomous decisions about its actions. We have tested this architecture by successfully reproducing a developmental psychology experiment [1] that aimed to evaluate the degree of experimental subjects' ToM through a sticker finding game, obtaining consistent results.

In the future, we plan to expand this architecture to make it progressively more general-purpose: thanks to the flexibility of BNs it is straightforward to reorganize nodes and edges to represent a wider range of real-life situations. As an example, it would be possible to take into account the contemporary influence of two or more informants, similarly to what has been done by [25]. Other fields of interest are elderly care [26], robotic-assisted surgery [27], joint-action HRI in the performing arts [28] and autonomous driving [29]. We also plan to use this model in a scenario where trust estimation and intention reading will generate and modulate collaborative behaviour between humans and robots.

## Supplementary Material

Video

## Data accessibility

Source code for this project is available in a GitHub public repository: https://github.com/samvinanzi/VanderBOT [30].
